# Prediabetes and Risk of All-Cause and Cause-Specific Mortality: A Prospective Study of 114 062 Adults in Mexico City

**DOI:** 10.1210/clinem/dgaf225

**Published:** 2025-04-10

**Authors:** Carlos Alberto Fermín-Martínez, Omar Yaxmehen Bello-Chavolla, César Daniel Paz-Cabrera, Daniel Ramírez-García, Jerónimo Perezalonso-Espinosa, Luisa Fernández-Chirino, Arsenio Vargas-Vázquez, Juan Pablo Díaz-Sánchez, Padme Nailea Méndez-Labra, Alejandra Núñez-Luna, Martín Roberto Basile-Alvarez, Paulina Sánchez-Castro, Fiona Bragg, Louisa Gnatiuc Friedrichs, Diego Aguilar-Ramírez, Jonathan R Emberson, Jaime Berumen, Pablo Kuri-Morales, Roberto Tapia-Conyer, Jesus Alegre-Díaz, Jacqueline A Seiglie, Neftali Eduardo Antonio-Villa

**Affiliations:** Research Division, Instituto Nacional de Geriatría, Mexico City 10200, Mexico; MD/PhD (PECEM) Program, Facultad de Medicina, Universidad Nacional Autónoma de México, Mexico City 04360, Mexico; Research Division, Instituto Nacional de Geriatría, Mexico City 10200, Mexico; Research Division, Instituto Nacional de Geriatría, Mexico City 10200, Mexico; Especialidad en Medicina Preventiva, Instituto Nacional de Salud Pública, Mexico City 14080, Mexico; Research Division, Instituto Nacional de Geriatría, Mexico City 10200, Mexico; Facultad de Medicina, Universidad Nacional Autónoma de México, Mexico City 04360, Mexico; Research Division, Instituto Nacional de Geriatría, Mexico City 10200, Mexico; MD/PhD (PECEM) Program, Facultad de Medicina, Universidad Nacional Autónoma de México, Mexico City 04360, Mexico; Research Division, Instituto Nacional de Geriatría, Mexico City 10200, Mexico; Clinical Trial Service Unit and Epidemiological Studies Unit, Nuffield Department of Population Health, University of Oxford, Oxford OX3 7LF, UK; MD/PhD (PECEM) Program, Facultad de Medicina, Universidad Nacional Autónoma de México, Mexico City 04360, Mexico; Research Division, Instituto Nacional de Geriatría, Mexico City 10200, Mexico; MD/PhD (PECEM) Program, Facultad de Medicina, Universidad Nacional Autónoma de México, Mexico City 04360, Mexico; Research Division, Instituto Nacional de Geriatría, Mexico City 10200, Mexico; MD/PhD (PECEM) Program, Facultad de Medicina, Universidad Nacional Autónoma de México, Mexico City 04360, Mexico; Research Division, Instituto Nacional de Geriatría, Mexico City 10200, Mexico; Facultad de Medicina, Universidad Nacional Autónoma de México, Mexico City 04360, Mexico; Research Division, Instituto Nacional de Geriatría, Mexico City 10200, Mexico; Facultad de Medicina, Universidad Nacional Autónoma de México, Mexico City 04360, Mexico; Research Division, Instituto Nacional de Geriatría, Mexico City 10200, Mexico; Facultad de Medicina, Universidad Nacional Autónoma de México, Mexico City 04360, Mexico; Clinical Trial Service Unit and Epidemiological Studies Unit, Nuffield Department of Population Health, University of Oxford, Oxford OX3 7LF, UK; Clinical Trial Service Unit and Epidemiological Studies Unit, Nuffield Department of Population Health, University of Oxford, Oxford OX3 7LF, UK; Clinical Trial Service Unit and Epidemiological Studies Unit, Nuffield Department of Population Health, University of Oxford, Oxford OX3 7LF, UK; Clinical Trial Service Unit and Epidemiological Studies Unit, Nuffield Department of Population Health, University of Oxford, Oxford OX3 7LF, UK; Facultad de Medicina, Universidad Nacional Autónoma de México, Mexico City 04360, Mexico; Instituto Tecnológico y de Estudios Superiores de Monterrey, Monterrey 64700, Mexico; Facultad de Medicina, Universidad Nacional Autónoma de México, Mexico City 04360, Mexico; Experimental Research Unit, Faculty of Medicine, National Autonomous University of Mexico, Mexico City 06720, Mexico; Diabetes Unit, Massachusetts General Hospital, Harvard Medical School, Boston, MA 02114, USA; Department of Medicine, Harvard Medical School, Boston, MA 02114, USA; Department of Endocrinology, Instituto Nacional de Cardiología Ignacio Chávez, Mexico City 14080, Mexico

**Keywords:** prediabetes, mortality, observational study, Mexican population

## Abstract

**Background:**

Prediabetes has been associated with increased all-cause and cardiovascular mortality. However, no large-scale studies have been conducted in Mexico or Latin America examining these associations.

**Methods:**

We analyzed data from 114 062 adults without diabetes (diagnosed or undiagnosed) from the Mexico City Prospective Study. Participants were followed until January 1, 2021, for cause-specific mortality. We defined prediabetes according to the American Diabetes Association (ADA; HbA1c ≥ 5.7% to <6.5%) and the International Expert Committee (IEC; HbA1c ≥ 6.0 to <6.5%) definitions. Cox regression adjusted for confounders was used to estimate all-cause and cause-specific mortality rate ratios (RR) for deaths occurring at ages 35 to 74 years associated with prediabetes.

**Results:**

After median 18.4 (IQR 17.6-19.7) years of follow-up, individuals with prediabetes had higher risk of all-cause mortality at ages 35 to 74 compared to those without prediabetes (RR 1.13 [1.07-1.20] for ADA-defined and 1.27 [1.17-1.38] for IEC-defined prediabetes), as well as higher risk of cardiovascular (RR 1.23 [1.11-1.37] and 1.44 [1.24-1.67], respectively), renal (RR 1.33 [1.06-1.66] and 1.62 [1.18-2.23], respectively), and acute diabetic deaths (RR 2.62 [1.75-3.93] and 3.42 [2.09-5.61], respectively). The absolute excess risk associated with ADA-defined prediabetes at ages 35 to 74 accounted for 7% of cardiovascular, 9% of renal, and 31% of acute diabetic deaths. IEC-defined prediabetes accounted for 4%, 5% and 14% of cardiovascular, renal, and acute diabetic deaths. Prediabetes-associated excess mortality risks were, at least in part, explained by adiposity.

**Conclusion:**

Prediabetes is a significant risk factor for all-cause, cardiovascular, renal, and acute diabetic deaths in Mexican adults. Early identification and timely management of prediabetes among individuals at risk of this condition could reduce premature mortality in this population.

Although the definition of prediabetes is the subject of continuous debate, it is well-recognized as a risk factor for development of type 2 diabetes and long-term adverse cardiometabolic outcomes (1-3). Prediabetes is an early-stage indicator of glycemic dysregulation, and individuals with prediabetes often present with other traits of the metabolic syndrome (ie, dysfunctional adipose tissue accumulation, dyslipidemia, insulin resistance) (4, 5). Despite its pathophysiological relevance and the overwhelming increase in the incidence of metabolic syndrome in susceptible populations, routine screening for prediabetes is disputed, in part due to a lack of consensus on how it should be defined (1). Growing evidence supports that beyond its well-known association with progression to diabetes, prediabetes also increases the risk of cardiovascular disease and mortality (3). Nevertheless, data on prediabetes-associated complications beyond progression to diabetes are limited, particularly in low- and middle-income countries (LMICs) (6).

In Mexico, the prevalence of diabetes has increased at an alarming rate in recent decades (7). This trend can be attributed to a complex interplay of underlying genetic risk and changes in environmental factors (8-10), which have led to a steady increase in central obesity, impaired glucose tolerance and insulin resistance (11-14). We recently described trends in prediabetes prevalence in the Mexican population (7); we found that although lower cutoffs of glycemic markers may be more adequate for screening strategies, these cutoffs may not fully reflect the long-term health risk associated with prediabetes (15). Notably, data on the risk conferred by prediabetes for all-cause and cause-specific mortality in Mexico are lacking. In this report, we aim to examine prediabetes as a risk factor for all-cause and cause-specific mortality in Mexican adults previously enrolled in the Mexico City Prospective Study (MCPS) and followed long-term for cause-specific mortality.

## Methods

### Study Design and Participants

Details on recruitment and follow-up of participants in MCPS have been described previously (16). Briefly, participants living in 2 municipalities in Mexico City (Coyoacán and Iztapalapa) aged ≥35 years were invited to participate in the study between 1998 and 2004. The study was approved by Ethics Committees at the Mexican Ministry of Health, the Mexican National Council for Science and Technology, and the University of Oxford, UK. All participants provided written informed consent.

### Data Collection

Sociodemographic, health-related, and lifestyle information were collected using an electronic questionnaire by trained nurses. All participants had their height, weight, hip circumference, waist circumference, and sitting blood pressure measured using calibrated instruments and standard protocols. A non-fasting venous blood sample was obtained and glycosylated hemoglobin (HbA1c) levels were measured using a validated high-performance liquid chromatography method (17) on HA-8180 analyzers with calibrators traceable to International Federation of Clinical Chemistry (IFCC) standards (18), and were reported in units of mmol/mol. To convert HbA1c to percent units according to the Diabetes Control and Complications Trial (DCCT) standard, we used the following formula:


HbA1c(DCCT)=[HbA1c(IFCC)*0.09148]+2.152

HbA1c defined in percentage units were used to define thresholds for prediabetes definitions as described below. (Note that with the above equation a glycosylated hemoglobin of 42 mmol/mol maps to 5.99% and therefore into the <6% category.) Metabolomic profiles in plasma samples were analyzed using nuclear magnetic resonance (NMR) spectroscopy on the Nightingale Health platform (19).

### Mortality Follow-Up

Participants were followed for cause-specific mortality through probabilistic linkage (based on name, age, and sex) to the Mexican electronic death registry. Death registration in Mexico City is reliable and complete, with nearly all deaths medically certified (20). Diseases recorded on death certificates are coded using the International Statistical Classification of Diseases and Related Health Problems, Tenth Revision, with subsequent review by study clinicians (unaware of baseline information) to recode, where necessary, the underlying cause of death. Participant deaths for the present study were tracked up to January 1, 2021. We grouped deaths as cardiac (mainly ischemic heart disease), cerebrovascular, other vascular (mainly peripheral arterial disease and thromboembolism), renal, acute diabetic crises (diabetic coma or ketoacidosis), hepatobiliary (mainly cirrhosis), other gastrointestinal (mainly peptic ulcer and gastrointestinal infections), neoplastic, respiratory (mainly pneumonia and chronic obstructive pulmonary disease), and external, ill-defined, or other causes of death. Full details on ICD-10 codes for individual causes of death are reported in Supplementary Material (21).

### Prediabetes and Diabetes Definitions

Prediabetes was defined according to the HbA1c cutoffs recommended by the American Diabetes Association (ADA) (HbA1c 5.7%-6.4%) (22) and by the International Expert Committee (IEC, HbA1c 6.0%-6.4%) (23). We excluded individuals with previously diagnosed diabetes (self-reported medical diagnosis or use of glucose-lowering pharmacotherapy) and individuals with undiagnosed diabetes (HbA1c ≥ 6.5% in someone without previously diagnosed diabetes). To explore the mortality risks in the population with HbA1c levels between the ADA and IEC cutoff points, additional analyses categorized participants according to HbA_1_c levels <5.7%, ≥ 5.7% to <6.0%, and ≥6.0% to <6.5%.

### Statistical Analysis

We limited our analysis to the population with complete data on HbA1c, mortality, and covariates. To limit the potential effects of reverse causation, we excluded those with self-reported chronic comorbidities at baseline (ischemic heart disease, stroke, chronic kidney disease, chronic obstructive pulmonary disease, cirrhosis, or cancer) (24). For the remaining participants (study population), we evaluated the prevalence of ADA and IEC-defined prediabetes separately by age (in 5-year groups) and sex. Uniformly age- and sex-standardized death rates were estimated for those with and without ADA and IEC-defined prediabetes, as well as for the complementary HbA1c categories (above), and reported as events per 1000 person-years.

The primary analyses were restricted to deaths at ages 35 to 74 years (ie, deaths one might consider to be “premature”), consistent with previous MCPS analyses (24). Analyses of deaths at ages 75 to 84 were also performed to disaggregate the effect of prediabetes on older populations. Participants who did not die from the cause under study were censored at the earliest of death from an alternative cause, the end of the risk period under consideration (eg, age 75 years for analyses of risk during ages 35-74 years), or the end of follow-up for mortality (January 1, 2021). Cox proportional hazards regression was used to estimate mortality rate ratios (RRs, estimated from the Cox hazard ratios) for all-cause and cause-specific mortality associated with prediabetes and HbA1c levels, and we subdivided the follow-up time of each participant in 5-year periods of age-at-risk using the Lexis expansion (*Epi* R package) (25, 26). The cause-specific log hazard ratio from a Cox model provides a useful summary statistic for the average log mortality rate ratio (RR) across different time periods of follow-up, even if the true RR varies between different follow-up periods (ie, even if there is nonproportionality of hazards). All models were stratified by sex and age-at-risk (in 5-year groups), and progressively adjusted for confounders in the following order: (i) municipality of residence (Coyocán or Iztapalapa) (region-level variations in population characteristics have been known to influence mortality related to cardiometabolic diseases in Mexico) (27); and (ii) education level (university/college, or other), physical activity (none, regular), smoking (never, former, current), and alcohol consumption (never, former, current). In additional analyses, we further adjusted for adiposity markers (body mass index [BMI] and waist-to-height ratio [WHtR], which could confound and/or be a common cause of prediabetes and mortality), as well as blood pressure (systolic and diastolic) and blood lipids measured by NMR (low-density lipoprotein cholesterol, high-density lipoprotein cholesterol, and triglycerides), which may be mediators of any association. Subgroup analyses estimated mortality RRs separately by age-at-risk and sex and across strata of BMI (<25 vs ≥25 kg/m^2^) and waist circumference (men: < 90 vs ≥90 cm; women: < 80 vs ≥80 cm). The excess mortality attributable to prediabetes (in a population without previously diagnosed or undiagnosed diabetes) was estimated by applying the cause-specific mortality RRs to the number of deaths among those with prediabetes. All analyses were conducted using R (version 4.4.2).

### Role of Funding Sources

The funders had no role in study design, data collection, data analysis, data interpretation, or writing of the report.

## Results

Of 159 517 participants who were originally recruited, 45 455 (28.5%) were excluded from the present analyses. These comprised 29 948 (18.8%) with diagnosed or undiagnosed diabetes at recruitment, a further 9056 (5.7%) with missing data on HbA1c or covariates, or with uncertain mortality linkage, an additional 5077 (3.2%) with chronic comorbidities at baseline, and 1374 (0.9%) aged ≥85 years at recruitment (Supplementary Figure 1) (21). Of the remaining 114 062 participants, 108 657 (95.3%) were aged 35 to 74 years (Table 1) and 5405 (4.7%) were aged 75 to 84 years at recruitment (Supplementary Table 1) (21).

**Table 1.** Baseline characteristics of 108 657 men and women aged 35 to 74 without previously diagnosed or undiagnosed diabetes or other chronic disease at recruitment

	All participants N = 108 657	HbA1c, %		
		<5.7 N = 80 301	≥5.7 to <6.0 N = 21 272	≥6.0 to <6.5 N = 7084
Age, years	49 (10)	47 (10)	52 (11)	54 (10)
HbA1c, %	5.45 (0.37)	5.29 (0.26)	5.83 (0.10)	6.22 (0.12)
Female sex, n (%)	73 586 (68%)	53 995 (67%)	14 611 (69%)	4980 (70%)
Residence in Coyoacán, n (%)	44 632 (41%)	36 846 (46%)	5926 (28%)	1860 (26%)
University/college educated, n (%)	19 304 (18%)	16 183 (20%)	2447 (12%)	674 (9.5%)
Smoking (%)				
Never	52 275 (48%)	37 817 (47%)	10 728 (50%)	3730 (53%)
Former	19 655 (18%)	14 404 (18%)	3961 (19%)	1290 (18%)
Current	36 727 (34%)	28 080 (35%)	6583 (31%)	2064 (29%)
Alcohol intake (%)				
Never	20 212 (19%)	14 406 (18%)	4321 (20%)	1485 (21%)
Former	6819 (6%)	5181 (6%)	1236 (6%)	402 (6%)
Current	81 626 (75%)	60 714 (76%)	15 715 (74%)	5197 (73%)
Regular leisure-time physical activity*^a^* (%)	24 900 (23%)	19 537 (24%)	4114 (19%)	1249 (18%)
Body mass index (kg/m^2^)	29.0 (4.9)	28.3 (4.7)	30.6 (5.1)	32.0 (5.3)
Waist-to-height ratio	0.60 (0.08)	0.59 (0.07)	0.63 (0.08)	0.66 (0.08)
Waist-Hip Ratio	0.89 (0.08)	0.89 (0.07)	0.91 (0.07)	0.92 (0.08)
Systolic blood pressure (mmHg)	126 (16)	125 (16)	129 (17)	132 (17)
Diastolic blood pressure (mmHg)	83 (11)	82 (11)	84 (11)	86 (11)
Lipid measurements*^b^* LDL cholesterol, mmol/L	2.42 (0.90)	2.40 (0.89)	2.49 (0.94)	2.44 (0.96)
HDL cholesterol, mmol/L	0.97 (0.41)	0.98 (0.40)	0.96 (0.43)	0.94 (0.43)
Triglycerides, mmol/L	1.49 (0.72)	1.44 (0.70)	1.59 (0.76)	1.69 (0.81)
Apolipoprotein A1, g/L	1.19 (0.41)	1.19 (0.40)	1.19 (0.44)	1.18 (0.44)
Apolipoprotein B, g/L	0.86 (0.39)	0.85 (0.38)	0.88 (0.42)	0.88 (0.43)

Results are presented as either mean (SD) or n (%). All baseline characteristics differed among HbA1c groups (*P* < 0.001, Kruskal-Wallis' test or Pearson's Chi-squared test); however, this reflects the large sample size rather than epidemiologically relevant differences.

^*a*^Regular physical activity was defined as at least 1 day per week.

^*b*^Blood lipids were measured using the Nightingale Health nuclear magnetic resonance platform.

**Figure 1.**
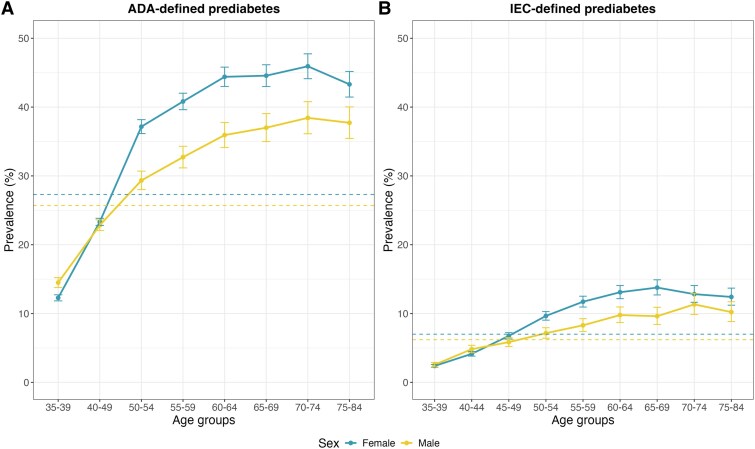
Prevalence of ADA- (A) and IEC-defined prediabetes (B) in 114 062 MCPS participants without previously diagnosed or undiagnosed diabetes, by age and sex. Dashed lines indicate overall prevalence of prediabetes for each definition stratified by sex. Analysis limited to 114 062 participants aged 35 to 84, and without previously diagnosed or undiagnosed diabetes (or other chronic disease) at recruitment. Abbreviations: ADA, American Diabetes Association; IEC, International Expert Committee.

Of the 108 657 participants aged 35 to 74 years (mean age 49 years) those in the highest HbA1c category (HbA1c ≥ 6.0% to <6.5%) were older, predominantly female, and had higher mean BMI, systolic blood pressure, and diastolic blood pressure than those with a lower HbA1c. Participants in the higher HbA1c category were less likely to reside in Coyoacán (the more affluent of the 2 study districts) than participants with a lower HbA1c (Table 1). A total of 28 356 individuals (26%) had ADA-defined prediabetes (HbA1c ≥ 5.7% to <6.5%) and 7084 (7%) had IEC-defined prediabetes (HbA1c ≥ 6.0% to <6.5%). The prevalence of prediabetes (regardless of definition) increased with age (at least up to age 70) and was higher in women than men (at least after age 40) (Fig. 1).

**Figure 2.**
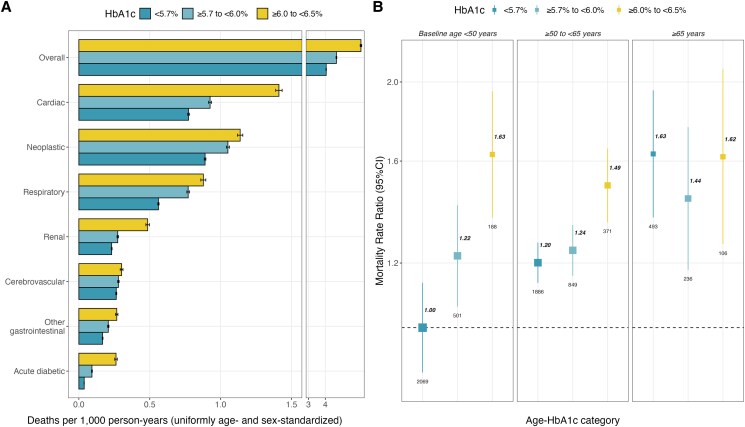
All-cause and cause-specific mortality at ages 35 to 74 years associated with prediabetes. Analysis limited to 108 657 participants aged 35 to 74 and without previously diagnosed or undiagnosed diabetes (or other chronic disease) at recruitment. (A) Uniformly age- and sex-standardized mortality rates per 1000 person-years according to HbA1c categories, error bars represent 95% CI. (B) Rate ratios with 95% CI for all-cause mortality associated with HbA1c categories, stratified by age at recruitment (<50 years, 50-64 years, or ≥65 years). Top bold numbers represent the mortality rate ratio, bottom numbers represent deaths recorded in each group; the size of the squares is proportional to the amount of statistical information. Models are stratified by sex and age-at-risk and adjusted for municipality, education level, physical activity, smoking, and alcohol intake. Each 95% CI reflects the variance of the log risk in that one group.

During a median of 18.4 (IQR 17.6-19.7) years' follow-up, there were 6699 deaths at ages 35 to 74 years (including 1750 from cardiovascular causes, 392 from renal causes, and 108 from acute diabetic crises) and 6923 deaths at ages 75 to 84 years. All-cause and cause-specific death rates (uniformly age- and sex-standardized) increased according to HbA1c category, with the highest death rates being observed in participants with HbA1c ≥ 6.0% to <6.5% (Fig. 2A). Given that prediabetes prevalence markedly increased with age, we explored the influence of baseline age and HbA1c categories. For participants aged <50 years at recruitment, HbA1c ≥ 6.0% to <6.5% was strongly associated with mortality risk, and this association was preserved for participants aged 50 to 64 years at recruitment. However, we identified that the graded increase in relative risk conferred by higher HbA1c levels was lost in older participants (Fig. 2B**)**. When stratified by sex, we observed broadly similar death rate ratios in men and women (Supplementary Figure 2) (21). Similar results were obtained when looking at deaths occurring at ages 75 to 84 years (Supplementary Figures 2 and 3) (21).

After adjustment for age, sex, and confounders, each 0.5% higher HbA1c was associated with 6% higher risk of death from any cause at ages 35 to 74 years (RR 1.06 [95% CI 1.02-1.10]) (Table 2, which also shows the RRs with more and with less adjustment) (21). Compared with individuals without prediabetes, those with ADA-defined prediabetes had 13% higher risk of death at ages 35 to 74 years (RR 1.13 [95% CI 1.07-1.20]) while those with IEC-defined prediabetes had 27% higher risk (RR 1.27 [95% CI 1.17-1.38]) (Table 2) (21). After additional adjustment for adiposity markers these RRs were reduced to 1.03 (0.97-1.09) and 1.14 (1.04-1.23), respectively (Table 2). Subsequent further adjustment for blood pressure and blood lipids had little further effect (21). Estimates were similar when including participants with other comorbidities at recruitment (Supplementary Table 2) (21). Among participants with normal BMI (<25 kg/m^2^), the prevalence of prediabetes was 2.3% for IEC-defined and 14.3% for ADA-defined prediabetes. In those with normal waist circumference, the prevalences were 1.8% and 12%, respectively. The effects of prediabetes on mortality did not differ significantly between participants with higher vs lower BMI (≥25 vs <25 kg/m^2^; *P* = 0.98) or between participants with higher vs lower waist circumference (men: ≥ 90 vs <90 cm; women: ≥ 80 vs <80 cm; *P* = 0.30).

**Table 2.** Association of HbA1c and ADA- and IEC-defined prediabetes with all-cause mortality at ages 35 to 74 years

Predictor	RR (95% CI)			
	Age-at-risk, sex, and district of residence	+ Education, physical activity, smoking, and alcohol intake	+ Adiposity measures (BMI and WHtR)	+ SBP, DBP, LDL-c, HDL-c, triglycerides
**Continuous (per 0.5% unit)**				
HbA1c (%)	1.07 (1.03, 1.12)	1.06 (1.02, 1.10)	0.98 (0.94, 1.02)	1.00 (0.96, 1.03)
**HbA1c category**				
*<5.7%*	1.00	1.00	1.00	1.00
*≥5.7% to <6.0%*	1.10 (1.03, 1.17)	1.07 (1.01, 1.14)	0.99 (0.93, 1.05)	1.01 (0.95, 1.07)
*≥6.0% to <6.5%*	1.34 (1.23, 1.46)	1.30 (1.20, 1.42)	1.13 (1.04, 1.23)	1.14 (1.05, 1.25)
**Prediabetes definition**				
ADA (*≥*5.7 to <6.5%)	1.16 (1.10, 1.22)	1.13 (1.07, 1.20)	1.03 (0.97, 1.09)	1.04 (0.99, 1.10)
IEC (*≥6.0 to <6.5%)*	1.30 (1.20, 1.41)	1.27 (1.17, 1.38)	1.14 (1.04, 1.23)	1.14 (1.05, 1.24)

Analyses limited to 108 657 participants aged 35 to 74 and without previously diagnosed or undiagnosed diabetes or other chronic disease at recruitment. All models are stratified by sex and age-at-risk in 5-year increments using the Lexis expansion.

Abbreviations: ADA, American Diabetes Association; BMI, body mass index; DBP, diastolic blood pressure; HDL-c, high-density lipoprotein cholesterol; IEC, International Expert Committee; LDL-c, low-density lipoprotein cholesterol; RR, mortality rate ratio; SBP, systolic blood pressure; WHtR, waist-to-height ratio.

Table 3 shows the distribution of the 6699 deaths at ages 35 to 74 years in participants with and without prediabetes (ADA and IEC definitions), as well as the cause-specific mortality RRs and their 95% CIs. Both ADA- and IEC-defined prediabetes were significantly associated with higher risk of cardiac death, all cardiovascular death, renal death, and death from an acute diabetic crisis (Supplementary Figure 4) (21). In addition, ADA-defined prediabetes was significantly associated with death from respiratory diseases. When examining deaths occurring at ages 75 to 84 years, cause-specific RRs tended to be slightly weaker, although IEC-defined prediabetes remained strongly predictive of both renal death and death from acute diabetic crises at these older ages (Supplementary Figure 5 and Supplementary Table 3) (21). Notably, each 0.5% increase in HbA1c was associated with higher risk of all-cause, cardiac, other vascular, cardiovascular, renal, acute diabetic, and respiratory deaths at 35 to 74 years when adjusted for age, sex, education, and lifestyle, while for deaths at 75 to 84 years each 0.5% increase in HbA1c was only clearly associated with higher risk of renal and acute diabetic deaths after adjustment for confounders. Further adjustment for lipids, blood pressure, and adiposity showed that each 0.5% increase in HbA1c was only associated with higher risk of acute diabetic and respiratory deaths at 35 to 74 years, and for acute diabetic deaths at 75 to 84 years (Supplementary Table 4) (21).

**Table 3.** Excess cause-specific mortality at ages 35 to 74 years associated with ADA and IEC definitions of prediabetes at recruitment

Cause	ADA definition				IEC definition			
	No. deaths		Death RR*^a^* (95% CI)	Attributable mortality*^b^* (%)	No. deaths		Death RR*^a^* (95% CI)	Attributable mortality*^b^* (%)
	No prediabetes	Prediabetes			No prediabetes	Prediabetes		
All causes	4448	2251	1.13 (1.07-1.20)	4	6034	665	1.27 (1.17-1.38)	2
Cardiac	770	448	1.27 (1.12-1.43)	8	1071	147	1.54 (1.29-1.84)	4
Cerebrovascular	251	136	1.09 (0.88-1.37)	3	354	33	0.97 (0.67-1.39)	0
Other vascular	84	61	1.51 (1.05-2.15)	14	121	24	2.11 (1.33-3.34)	9
All cardiovascular	1105	645	1.23 (1.11-1.37)	7	1546	204	1.44 (1.24-1.67)	4
Renal	244	148	1.33 (1.06-1.66)	9	343	49	1.62 (1.18-2.23)	5
Acute diabetic crisis	54	54	2.62 (1.75-3.93)	31	86	22	3.42 (2.09-5.61)	14
Gastrointestinal	747	286	0.84 (0.72-0.96)	−5	928	105	1.31 (1.06-1.60)	2
Neoplastic	1045	506	1.09 (0.97-1.21)	3	1418	133	1.08 (0.90-1.29)	1
Respiratory	686	364	1.26 (1.10-1.44)	7	961	89	1.12 (0.90-1.41)	1
External/ill-defined/other	567	248	1.06 (0.91-1.25)	2	752	63	1.04 (0.80-1.35)	0

Abbreviations: ADA definition, HbA1c ≥ 5.7% to <6.5%; IEC definition = HbA1c ≥ 6.0% to <6.5%; RR, rate ratio.

^*a*^Mortality rate ratio estimates for those with vs without prediabetes at recruitment were stratified by sex and age-at-risk and adjusted for municipality, education level, physical activity, smoking, and alcohol intake. Analyses excluded data from participants with previously diagnosed or undiagnosed diabetes or other prior chronic disease.

^*b*^For each definition of prediabetes, the number of deaths attributable to the excess risk associated with it was calculated as D × (RR − 1)/RR, where D is the proportion of deaths in the prediabetes group and RR is the cause-specific mortality rate ratio for prediabetes vs no prediabetes.

Overall, between 35 and 74 years of age in the population without previously diagnosed or undiagnosed diabetes, the excess mortality risk associated with ADA-defined prediabetes accounted for 4% of all deaths, including 7% of cardiovascular deaths, 9% of renal deaths, and 31% of deaths from an acute diabetic crisis (Table 3). By contrast, the excess mortality risk associated with IEC-defined prediabetes accounted for 2% of all deaths, including 4% of cardiovascular deaths, 5% of renal deaths, and 14% of deaths from an acute diabetic crisis.

## Discussion

In this large study of Mexican adults, we explored the association of HbA1c-defined prediabetes with all-cause and cause-specific mortality. Overall, both ADA- and IEC-defined prediabetes were associated with a higher risk of all-cause mortality. The RRs associated with IEC-defined prediabetes (HbA1c ≥ 6% to <6.5%) were somewhat larger than those associated with ADA-defined prediabetes (HbA1c ≥ 5.7% to <6.5%). When considering cause-specific mortality, both definitions were associated with a higher risk of mortality from cardiovascular disease, kidney disease, and acute diabetic crises, but only the ADA definition was significantly associated with respiratory-related mortality, perhaps reflecting increased statistical power for this definition. These results support the view that prediabetes is a significant risk factor for mortality associated with cardiometabolic causes.

A recently updated meta-analysis of prospective cohorts reported a 13% higher risk of all-cause mortality when considering any definition of prediabetes (3). When examining definitions based on HbA1c, the IEC definition yielded a 21% increase in risk, while the ADA definition was not significantly associated with mortality (3). In the present study, we found that prediabetes was significantly associated with higher risk of premature death from cardiovascular disease, kidney disease, and acute diabetic crises irrespective of the definition used. This is in line with previous reports from other populations although, unlike previous reports, we did not find any significant association with cancer death (3, 28-30). Notably, estimates of mortality associated with ADA and IEC definitions were reasonably consistent, with differences in attributable mortality being a function of prevalence more than associated mortality risks. In our study, both definitions of prediabetes were associated with mortality independently of structural determinants and lifestyle factors, highlighting the relevance of prediabetes across different risk profiles. The strength of the relationship between prediabetes and mortality was at least partially attenuated after adjusting for adiposity measures, reflecting the relevance of adiposity as a causal determinant of prediabetes and of many causes of mortality. This clearly does not diminish the importance of prediabetes as a risk factor for mortality in this population, and the associations of prediabetes with mortality did not differ much across strata of BMI or waist circumference.

Expanding prediabetes screening has been challenging, given that preventive efforts should not only be aimed at preventing progression to type 2 diabetes but also risk of all-cause and cause-specific mortality (2, 3, 22). Our findings support the view that the IEC definition of prediabetes could be more useful at identifying individuals at the highest risk for cause-specific mortality compared to the ADA definition, at the same time limiting potential adverse impacts on individuals and healthcare systems of labeling a large proportion of the population with a diagnosis of prediabetes (1). However, selecting the optimal point for screening should also balance the relevance of additional outcomes, perhaps most importantly type 2 diabetes. Although trial evidence supporting a mortality benefit of prediabetes prevention is lacking, long-term follow-up of participants in trials of type 2 diabetes prevention among individuals with prediabetes have shown reductions in vascular diseases and mortality (31). However, these trials have not been performed in Latin American populations, in which sociodemographic and structural factors play a prominent role in cardiometabolic health (32). Further studies are warranted to translate our findings into prevention strategies that could be implemented in this context.

Nonetheless, the significance of prediabetes as a risk factor for all-cause and cause-specific mortality lies mainly within its high prevalence worldwide (33). Previous efforts from our group reported an increasing prevalence of HbA1c-defined prediabetes in Mexico between 2016 and 2022 (7). Although the lack of representativeness of the MCPS cohort to the overall Mexican population limits the generalizability of our prevalence estimates, the predominantly urban MCPS sample in the current report yielded a high prevalence of prediabetes (one-quarter of those without diabetes had ADA-defined prediabetes), which is similar to that estimated in a previous cross-sectional study of adults in Mexico City (26% of the adult population as a whole) (34).

In the present analysis, we observed that among those without diabetes, 7% of premature cardiovascular deaths, 9% of premature renal deaths and 31% of premature acute diabetic deaths could be attributed to the long-term hazards associated with ADA-defined prediabetes. Interestingly, we also identified that the magnitude of the risk ratio of prediabetes for all-cause and cause-specific mortality decreased at older ages, being consistent with findings from the ARIC study, which identified that regression to normoglycemia from prediabetes was more common in this population, potentially contributing to the decrease in at-risk population over time (35). However, epidemiological studies assessing these exposures on aging populations are currently lacking, and further research is needed to elucidate the effect of prediabetes in older adults.

Our study has several strengths, including its large sample size, prospective design, and prolonged follow-up period, which allowed us to evaluate long-term prediabetes-related outcomes compared with previous studies. However, we also acknowledge some limitations which should be considered to adequately frame our results. First, by only using HbA1c-based definitions of prediabetes, we were unable to assess separately the mortality risks associated with impaired fasting glucose and/or glucose intolerance; this may limit our ability to identify differential risk of adverse outcomes based on the specific definition employed (4, 5, 36). Moreover, since prediabetes status was ascertained from HbA1c levels in blood samples collected at recruitment and analyzed >10 years later, it was not possible to assess the relevance of age at diagnosis of prediabetes for mortality risks. Nevertheless, by exploring both ADA and IEC HbA1c-based definitions we contribute valuable insights to inform debate regarding the most appropriate definition of prediabetes for predicting future cardiometabolic disease risks. Prediabetes has also been recognized to increase the risk of a cluster of multiple chronic comorbidities, which may underlie the associations observed for mortality (37). To isolate the effect of prediabetes, we excluded individuals with comorbidities from our main analyses and focused on premature death to reduce the potential influence of aging in our estimations. Despite these adjustments, we cannot rule out the possibility of residual confounding or reverse causality in our findings. Furthermore, due to study design, we did not have prospective data to identify participants with prediabetes who developed diabetes or other nonfatal events during follow-up. This prevented us from analyzing the relevance of prediabetes for mortality and cardiometabolic risks independent of its association with increased risk of progression to diabetes, an area which will require further evaluation with longitudinal assessments. HbA1c measurements have been known to be impacted over time by individual-level factors, including chronic diseases, kidney, and liver function (38); this effect may have been mitigated in the present study by excluding individuals with comorbidities. However, we were unable to account for all potentially relevant factors, such as anemia and related states (eg, iron and/or vitamin B deficiencies). Lastly, we acknowledge that MCPS data, derived from 2 districts in Mexico City, is not nationally representative of the Mexican population. This includes a population structure biased toward women (since women were more likely to be at home during standard working hours when fieldworkers visited) (16). Studies aiming to estimate absolute risks require a representative population sample. However, large-scale prospective studies of non-representative cohorts of individuals with heterogeneity of exposure status, such as MCPS, can provide reliable estimates of the associations of risk factors with disease that are widely generalizable (39, 40). Moreover, since death rates among men in MCPS are approximately twice as high as among women, this ensures inclusion of large and broadly similar numbers of deaths in men and women.

In this Mexican population, prediabetes was significantly associated with higher risks of cardiovascular, renal, and acute diabetic mortality. Given these risks associated with prediabetes, coupled with the high prevalence of prediabetes in Mexican adults, population-wide screening strategies to facilitate the timely diagnosis of prediabetes, alongside implementation of strategies to prevent and delay associated cardiometabolic diseases and related mortality, should be considered. However, further work is needed to identify the appropriate outcome-driven definitions of prediabetes to promote efficient screening and prevention strategies that minimize overdiagnosis and improve cardiometabolic outcomes.

## Data Availability

Data from the Mexico City Prospective Study are available to bona fide researchers. For more details, the study's Data and Sample Sharing policy may be downloaded (in English or Spanish) from https://www.ctsu.ox.ac.uk/research/mcps. Available study data can be examined in detail through the study's Data Showcase, available at https://datashare.ndph.ox.ac.uk/mexico/. Code and Supplementary Materials and Methods are available for reproducibility of results at https://github.com/oyaxbell/prediabetes_mcps/ (21).

## Rights Retention Statement

For the purposes of open access, the authors have applied a Creative Commons Attribution (CC BY) license to any Author Accepted Manuscript version arising.

## Abbreviations

ADA: American Diabetes Association
BMI: body mass index
HbA1c: glycosylated hemoglobin
IEC: International Expert Committee
MCPS: Mexico City Prospective Study
RR: rate ratio
